# Antioxidant and acetylcholinesterase-inhibitory properties of long-term stored medicinal plants

**DOI:** 10.1186/1472-6882-12-87

**Published:** 2012-07-07

**Authors:** Stephen O Amoo, Adeyemi O Aremu, Mack Moyo, Johannes Van Staden

**Affiliations:** 1 Research Centre for Plant Growth and Development, School of Life Sciences, University of KwaZulu-Natal Pietermaritzburg, Private Bag X01, Scottsville, 3209, South Africa

**Keywords:** Antioxidants, Acetylcholinesterase inhibition, Long-term storage, Medicinal plants, Radical scavenging activity

## Abstract

**Background:**

Medicinal plants are possible sources for future novel antioxidant compounds in food and pharmaceutical formulations. Recent attention on medicinal plants emanates from their long historical utilisation in folk medicine as well as their prophylactic properties. However, there is a dearth of scientific data on the efficacy and stability of the bioactive chemical constituents in medicinal plants after prolonged storage. This is a frequent problem in African Traditional Medicine.

**Methods:**

The phytochemical, antioxidant and acetylcholinesterase-inhibitory properties of 21 medicinal plants were evaluated after long-term storage of 12 or 16 years using standard *in vitro* methods in comparison to freshly harvested materials.

**Results:**

The total phenolic content of *Artemisia afra*, *Clausena anisata*, *Cussonia spicata*, *Leonotis intermedia* and *Spirostachys africana* were significantly higher in stored compared to fresh materials. The flavonoid content were also significantly higher in stored *A. afra*, *C. anisata*, *C. spicata*, *L. intermedia*, *Olea europea* and *Tetradenia riparia* materials. With the exception of *Ekebergia capensis* and *L. intermedia*, there were no significant differences between the antioxidant activities of stored and fresh plant materials as measured in the *β-*carotene-linoleic acid model system. Similarly, the EC_50_ values based on the 2,2-diphenyl-1-picrylhydrazyl (DPPH) free radical scavenging assay were generally lower for stored than fresh material. Percentage inhibition of acetylcholinesterase was generally similar for both stored and fresh plant material. Stored plant material of *Tetradenia riparia* and *Trichilia dregeana* exhibited significantly higher AChE inhibition than the fresh material.

**Conclusions:**

The current study presents evidence that medicinal plants can retain their biological activity after prolonged storage under dark conditions at room temperature. The high antioxidant activities of stable bioactive compounds in these medicinal plants offer interesting prospects for the identification of novel principles for application in food and pharmaceutical formulations.

## Background

The detrimental effects of oxidative stress to human tissues and cells caused by reactive oxygen species (ROS) arising from aging and disease pathogenesis is well documented. Though the human body has inherent antioxidative mechanisms to counteract the damaging effects of free radicals, there is often a need to use dietary and/or medicinal antioxidant supplements, particularly during instances of disease attack. An imbalance between ROS such as singlet oxygen, superoxide anion radical, hydroxyl radical and hydrogen peroxide, and the natural detoxification capacity of the body in favour of the oxidant molecules causes oxidative stress leading to cellular and DNA damage as well as oxidation of low-density lipoproteins [1,2]. Oxidative stress disorders caused by the actions of ROS are associated with many acute and chronic diseases such as inflammation and neurodegenerative conditions including Alzheimer’s disease (AD) [3]. Alzheimer’s disease, an age-related neurological disorder, is characterised by progressive loss of cognitive ability primarily memory loss, leading to dementia. The main strategy in the clinical treatment of AD involves the maintenance of adequate levels of acetylcholine (ACh) at neurotransmission sites [4]. Thus, the inhibition of acetylcholinesterase (AChE) prevents the hydrolysis of ACh thereby maintaining normal memory function. The consumption of antioxidants is highly correlated with lower incidences of AD [5,6]. As a result, the use of natural compounds with high levels of antioxidants has been proposed as an effective therapeutic approach for AD [5].

Against a background of growing concerns about the toxicity and side effects of many synthetic therapeutic agents, there has been a renewed interest globally, in the search for antioxidants and AChE inhibitory compounds from natural sources, particularly medicinal plants [1,2,7-14]. Medicinal plants have long been used to treat cognitive memory dysfunction symptoms [4,5,15-19]. The growing relevance of medicinal plants as possible sources for the discovery of novel antioxidant molecules is often based on their long historical utilisation in folk medicine, especially in developing countries. In addition, the recognised health benefits of medicinal plants emanate from their prophylactic properties [6]. Most notably, traditional practices in the Ayurvedic, Chinese and African medicinal systems are strongly based on prevention and the promotion of good health; hence plant extracts and herbal preparations are regularly consumed as rejuvenators, tonics and/or nutritional supplements [8]. Traditional medicine practitioners and gatherers often store plants before they are eventually consumed. However, there is a dearth of scientific data on the stability and efficacy of the bioactive compounds in medicinal plants after prolonged storage. In the present study, 21 commonly used South African medicinal plants (Table 1) were investigated for their phytochemical, antioxidant and AChE-inhibitory properties after 12 or 16 years storage in comparison to freshly harvested material. These plants are used in traditional medicine to prevent and/or treat pain-related ailments and infections [20-23]. Fresh materials were harvested from the same locations and season as the stored materials [21,23] to minimise any differences due to geographical and seasonal effects [24].

**Table 1:** Effect of long-term storage on the total iridoid, phenolic and flavonoid contents of 21 South African medicinal plants

**Plant name**	**Family**	**Voucher number**	**Plant part(s)**	**Total iridoids (μg HE/g DW)**		**Total phenolics (mg GAE/g DW)**		**Total flavonoids (mg CE/g DW)**	
				**Stored**	**Fresh**	**Stored**	**Fresh**	**Stored**	**Fresh**
*Acokanthera oppositifolia* (Lam.) Codd^δ^	Apocynaceae	A. Aremu 1 NU	Roots	264.6 ± 4.82 **	134.5 ± 5.51	7.5 ± 0.37 *	9.3 ± 0.44	4.8 ± 0.12 *	5.4 ± 0.17
*Artemisia afra* Jacq. ex Willd^#^	Asteraceae	S. Amoo 15 NU	Aerial parts	356.9 ± 22.72 ns	341.7 ± 19.97	28.5 ± 1.15 ns	25.8 ± 0.03	18.3 ± 0.65 ns	16.7 ± 0.34
*Artemisia afra* Jacq. ex Willd^δ^	Asteraceae	S. Amoo 15 NU	Aerial parts	195.1 ± 63.35 ns	341.7 ± 19.97	34.7 ± 1.79 **	25.8 ± 0.03	19.7 ± 0.87 *	16.7 ± 0.34
*Buddleja salviifolia* (L.) Lam^#^	Buddlejaceae	S. Amoo 16 NU	Leaves	60.8 ± 15.84 **	409.9 ± 13.77	9.0 ± 0.36 ***	20.0 ± 0.81	6.6 ± 0.28 ***	14.6 ± 0.32
*Buddleja salviifolia* (L.) Lam^#^	Buddlejaceae	S. Amoo 16 NU	Twigs	111.1 ± 9.64 **	400.3 ± 27.54	8.3 ± 0.25 ***	11.1 ± 0.24	5.0 ± 0.25 *	5.9 ± 0.11
*Clausena anisata* (Willd.) Hook. F. ex Benth^#^	Rutaceae	S. Amoo 18 NU	Leaves & Twigs	3019.6 ± 63.35 ns	3264.7 ± 96.40	31.3 ± 0.05 *	28.1 ± 0.99	11.7 ± 0.17 ***	7.6 ± 0.20
*Cussonia spicata* Thunb. ^#^	Araliaceae	S. Amoo 09 NU	Leaves	82.8 ± 39.25 ns	38.8 ± 11.71	11.4 ± 0.16 **	7.6 ± 0.69	9.1 ± 0.53 ***	3.4 ± 0.27
*Dombeya rotundifolia* Hochst. ^#^	Malvaceae	S. Amoo 11 NU	Leaves	7076.6 ± 177.64 **	9499.6 ± 117.75	45.3 ± 0.89 ns	47.3 ± 1.94	29.7 ± 3.05 ns	35.4 ± 0.87
*Ekebergia capensis* Sparrm^δ^	Meliaceae	S. Amoo 23 NU	Leaves & Twigs	547.6 ± 22.03 ***	2221.5 ± 53.02	31.7 ± 1.29 ***	44.9 ± 0.78	22.8 ± 1.25 ns	26.0 ± 0.29
*Leonotis intermedia* Lindl.^δ^	Lamiaceae	S. Amoo 08 NU	Leaves	56.0 ± 1.38 *	72.5 ± 2.75	15.1 ± 0.57 **	11.6 ± 0.23	12.1 ± 0.38 ***	6.8 ± 0.10
*Leonotis leonurus* (L.) R.Br.^*δ*^	Lamiaceae	S. Amoo 12 NU	Leaves	51.8 ± 1.38 ns	171.0 ± 30.99	10.5 ± 0.22 ***	18.2 ± 0.76	6.6 ± 0.23 ***	10.3 ± 0.01
*Merwilla plumbea* (Lindl.) Septa^δ^	Hyacinthaceae	S. Amoo 21 NU	Bulbs	64.2 ± 8.26 ns	207.5 ± 75.74	7.8 ± 0.29 **	9.8 ± 0.25	1.4 ± 0.09 ns	1.7 ± 0.37
*Ocotea bullata* (Burch.) Baill.^δ^	Lauraceae	S. Amoo 13 NU	Bark	3060.9 ± 121.19 **	6112.6 ± 207.95	32.7 ± 0.82 **	46.4 ± 2.00	18.4 ± 0.62 ***	26.8 ± 0.50
*Olea europaea* L.^#^	Oleaceae	S. Amoo 14 NU	Leaves	0 ns	283.2 ± 79.87	17.2 ± 0.41 *	18.7 ± 0.06	13.1 ± 0.31 ***	9.7 ± 0.28
*Pittosporum viridiflorum* Sims^#^	Pittosporaceae	S. Amoo 24 NU	Leaves & Twigs	63.6 ± 8.95 ns	194.4 ± 65.41	10.6 ± 0.20 ***	26.0 ± 0.91	5.3 ± 0.12 ***	15.6 ± 0.22
*Plumbago auriculata* Lam.^δ^	Plumbaginaceae	S. Amoo 06 NU	Leaves	9.8 ± 7.57 **	521.4 ± 50.95	7.6 ± 0.66 ***	15.0 ± 0.46	1.3 ± 0.15 **	5.5 ± 0.64
*Protorhus longifolia* (Bernh.) Engl.^δ^	Anacardiaceae	S. Amoo 19 NU	Leaves	1034.4 ± 47.51 **	7787.2 ± 290.57	51.8 ± 1.27 ***	114.4 ± 7.83	10.1 ± 0.65 ***	18.3 ± 0.10
*Solanum mauritianum S*cop.^δ^	Solanaceae	S. Amoo 07 NU	Leaves	71.1 ± 6.89 *	14.0 ± 11.71	8.0 ± 0.11 ***	13.9 ± 0.24	2.0 ± 0.21 ns	1.5 ± 0.05
*Spirostachys africana* Sond.^#^	Euphorbiaceae	S. Amoo 26 NU	Leaves & Twigs	553.8 ± 3.44 ns	527.6 ± 11.71	86.2 ± 1.91 **	69.1 ± 2.13	8.5 ± 0.09 ***	26.7 ± 0.57
*Synadenium copulare* (Boiss.) L.C. Wheeler^*δ*^	Euphorbiaceae	S. Amoo 25 NU	Leaves	11.9 ± 11.02 ns	273.6 ± 71.61	8.5 ± 0.37 ***	15.2 ± 0.33	4.2 ± 0.15 ns	4.3 ± 0.15
*Tetradenia riparia* (Hochst.) Codd^δ^	Lamiaceae	S. Amoo 20 NU	Leaves	46.3 ± 9.64 *	0	6.1 ± 0.20 ns	7.2 ± 0.38	2.7 ± 0.08 ***	1.5 ± 0.02
*Trichilia dregeana* Sond.^δ^	Meliaceae	S. Amoo 22 NU	Leaves & Twigs	431.9 ± 16.53 ns	412.0 ± 50.27	34.4 ± 10.26 ns	32.6 ± 1.17	8.7 ± 0.61 ***	20.2 ± 0.19
*Ziziphus mucronata* Willd.^#^	Rhamnaceae	S. Amoo 17 NU	Leaves	314.2 ± 37.87 ns	412.7 ± 22.03	23.6 ± 1.61 **	33.4 ± 0.62	7.1 ± 0.10 ***	9.0 ± 0.09
*Ziziphus mucronata* Willd.^δ^	Rhamnaceae	S. Amoo 17 NU	Leaves	90.4 ± 1.38 **	412.7 ± 22.03	19.7 ± 0.42 ***	33.4 ± 0.62	6.9 ± 0.34 **	9.0 ± 0.09

ns = not significant; *P* = 0.05 (*); *P* = 0.01 (**); *P* = 0.001 (***).

HE = harpagoside equivalents; GAE = gallic acid equivalents; CE = catechin equivalents.

^δ^ = Voucher number of plant material stored for 16 years was as described by Jäger *et al*. (1996); ^#^ = Voucher number of plant material stored for 12 years was as described by McGaw *et al*. (2000).

*Merwilla plumbea* (Lindl.) Speta was formerly known as *Scilla natalensis* Planch.

## Methods

### Chemicals and reagents

Acetylcholine iodide, AChE from electric eel (type VI-S lyophilized powder), *β*-carotene, 2,2-diphenyl-1-picrylhydrazyl (DPPH), 5,5-dithiobis-2-nitrobenzoic acid (DTNB), galanthamine, gallic acid, catechin and linoleic acid were obtained from Sigma-Aldrich (Steinheim, Germany); butylated hydroxytoluene (BHT) from BDH Chemicals Ltd. (Poole, England); and harpagoside from Extrasynthèse (France). All chemicals and reagents used were of analytical grade.

### Plant material and preparation of extracts

Table 1 shows the scientific names, and voucher specimen numbers of the evaluated plant materials. Following oven-drying at 50 °C, plant materials were stored at room temperature (25 °C) in brown paper bags in the dark for 12 or 16 years. Fresh plant materials collected from the same locations and season as the stored ones were similarly oven-dried at 50 °C. The plants were identified by Dr C. Potgieter and voucher specimens deposited in The Bews Herbarium, University of KwaZulu-Natal, Pietermaritzburg, South Africa.

Dried plant materials were ground to fine powders and extracted with 50% methanol at 20 ml/g in a sonication bath containing ice-cold water for 1 h for antioxidant and AChE assays. Extracts were then filtered through Whatman No. 1 filter paper, concentrated *in vacuo* at 40 °C and completely air-dried at room temperature in glass vials.

The extraction method described by Makkar [25] was used for phytochemical analysis. Dried plant materials, ground to fine powders (0.2 g), were extracted with 50% aqueous methanol (10 ml) in a sonication bath containing ice-cold water for 20 min. The extracts were then centrifuged at approximately 3000 U/min for 5 min using a Hettich Universal 1200 01 Centrifuge. The supernatants were collected and kept on ice for phytochemical analysis.

### Phytochemical analysis

Total iridoid content of the plant material was quantified using the method described by Levieille and Wilson [26]. The calibration curve was plotted using harpagoside as the standard. Total iridoid content for each plant material was expressed in μg harpagoside equivalents (HE) per g dry weight (DW).

For the determination of total phenolic content, the Folin & Ciocalteu [27] method was used with slight modifications [28]. Gallic acid was used as the standard for plotting the calibration curve. Total phenolic content was expressed in mg gallic acid equivalents (GAE) per g DW.

The flavonoid content of the plant materials were quantified using the aluminium chloride colorimetric method [29]. Catechin was used as a standard for the calibration curve. Flavonoid content was expressed in mg catechin equivalents (CE) per g DW.

The butanol-HCl method [25] was used to quantify condensed tannin (proanthocyanidin) content of the plant materials. Condensed tannins (% in dry matter) were expressed as leucocyanidin equivalents were calculated using the formula:

(1) $$Condensed\;tannins\;\left(\%\;dry\;matter\right)=\left(\frac{A_{550nm}\times 78.26\times Dilution\;factor}{\%\;dry\;matter}\right)\times 100$$

where *A*_550nm_ is the absorbance of the sample at 550 nm. The formula assumes that the effective $E_{550}^{1\%}$ of leucocyanidin is 460 [30].

Free gallic acid and gallotannin contents were evaluated using the rhodanine assay [25,31]. The calibration curves were plotted using gallic acid as a standard. Free gallic acid and gallotannin contents were expressed in μg GAE per g DW.

## Antioxidant activity

### DPPH free radical scavenging activity

The DPPH assay [32] was used to evaluate the free radical scavenging activity of the plant extracts. Methanol was used as a negative control while ascorbic acid and BHT were used as positive controls. Any absorbance due to extract colour was removed by including a background solution with methanol in place of DPPH solution for each extract. Each sample was evaluated in triplicate. The radical scavenging activity (RSA) was calculated using the equation:

(2) $$RSA\;\left(\%\right)=\left[1-\left(\frac{A_{\mathrm{extract}}-A_{\mathrm{background}}}{A_{\mathrm{control}}}\right)\right]\times 100$$

where *А*_extract_*A*_background_ and *A*_control_ are the absorbance readings of the extract, background solution and negative control, respectively at 517 nm. The EC_50_, which is the extract concentration required to scavenge 50% of DPPH free radical, was determined for each extract. Antioxidant activity index (AAI) for each extract was calculated using the equation [33]:

(3) $$AAI=\frac{Final\;DPPH\;concentration}{EC_{50}}$$

### *β*-Carotene-linoleic acid model system

The assay was done following the method described by Moyo et al. [34]. Methanol and BHT were used as negative and positive controls, respectively. Each sample was prepared in triplicate. The plant extracts and BHT were evaluated at a final assay concentration of 200 μg/ml. Antioxidant activity (%), measured at *t* = 120 min, was calculated using the following equations:

(4) $$Rate\;of\;\beta -carotene\;bleaching=In\;\left(\frac{A_{t=0}}{A_{t=t}}\right)\times \frac{1}{t}$$

(5) $$Antioxidant\;activity\;\left(\%\right)=\left(\frac{Rcontorl-Rsample}{Rcontrol}\right)\times 100$$

where A_t = 0_ is the initial absorbance at *t* = 0 min, A_t = t_ is the absorbance at time *t* = 120 min, t = 120 min and *R* is the rate of *β*-carotene bleaching.

### Acetylcholinesterase inhibitory activity

The AChE assay was performed using the colorimetric method [35]. Each extract was evaluated in triplicate at a final assay concentration of 1.0 mg/ml. Galanthamine at a final assay concentration of 20 μM was used as a positive control. The rate of reaction was calculated for each of the plant extracts, the blank (methanol) and positive control (galanthamine). The percentage inhibition by each plant extract was calculated using the formula:

(6) $$AChE\;inhibition\;\left(\%\right)=\left(1-\frac{Sample\;reaction\;rate}{Blank\;reaction\;rate}\right)\times 100$$

### Data analysis

The levels of significant difference between the mean values of stored and fresh plant materials were determined using the *t*-test (SigmaPlot version 8.0). Regression analysis and the determination of EC_50_ values were done using GraphPad Prism software (version 4.03).

## Results and discussion

### Phytochemical analysis

The effects of long-term storage on the total iridoid, phenolic and flavonoid content of the plant materials evaluated are presented in Table 1. Of the 21 fresh and stored plant materials evaluated, the levels of total iridoid present in nine plants were significantly higher in fresh compared to the stored plant materials. The total iridoid contents of stored materials in *Acokanthera oppositifolia*, *Solanum mauritanum* and *Tetradenia riparia* were significantly higher than those of fresh ones. There was no significant difference between the iridoid content of fresh and stored plant materials in approximately 50% of the evaluated plants.

The total phenolic contents of *Artemisia afraClausena anisataCussonia spicataLeonotis intermedia* and *Spirostachys africana* stored materials were significantly higher than in freshly collected material. With the exceptions of *A. afraD. rotundifoliaT. riparia* and *T. dregeana* (where there was no significant difference between the stored and fresh materials), the phenolic contents of the remaining 15 fresh plant materials were significantly higher than in the stored material. Similarly, a comparison of fresh material and herbarium specimens of three *Quillaja* species revealed non-significant differences in their phenolic constituents [36]. Remarkably, one of the tested herbarium specimens in the Bate-Smith [36] study was 100 years old.

The flavonoid content was significantly higher in stored *A. afraC. anisataC. spicataL. intermediaT. riparia* and *Olea europea* materials when compared to their corresponding fresh materials. It is noteworthy that the stored materials of the former four species had higher total phenolic contents than their fresh materials perhaps due to their higher flavonoid content compared to the fresh materials. Higher flavonoid contents were observed in 12 fresh plant materials when compared to their respective stored materials. Previous studies comparing the phenolic constituents of some *Dillenia* species showed differences in the flavonoid profiles of fresh and herbarium materials as some flavonoids were not detected in the latter [37]. The results suggested that some flavonoids are easily oxidised during the drying process [37].

Table 2 presents the condensed tannin, free gallic acid and gallotannin contents of both the stored and fresh materials of plant species evaluated in this study. No condensed tannins were detected in both fresh and stored materials of *A. oppositifoliaPittosporum viridiflorum* and *Merwilla plumbea*. With the exceptions of *Buddleja salviifolia* (leaves), *Plumbago auriculata* and *Ziziphus mucronata*, the condensed tannin content in the stored plant materials was either significantly higher or not different when compared to the fresh materials. Unlike the stored materials, no condensed tannins were detected in fresh material of *A. afraC. spicataL. intermediaLeonotis leonurus* and *O. europea*. Among the 21 species evaluated, free gallic acid was detected in 15 fresh and/or stored plant materials. In most cases, there was no significant difference in the free gallic acid contents of the fresh materials when compared to the stored ones. With the exceptions of *A. oppositifoliaA. afra* and *Ekebergia capensis*, the gallotannin content of the stored plant materials was either higher or not significantly different when compared to the fresh ones. It has been shown that phytochemical constituents of medicinal plants, such as alkaloids, flavonoids, volatile oils and amino acids are sufficiently stable to even be detected in herbarium specimens [38]. However, based on the results of the present study, the degree of stability of phenolic compounds seems to be species dependent.

**Table 2:** Effect of long-term storage on the condensed tannin, free gallic acid and gallotannin contents of 21 South African medicinal plants

**Plant name**	**Plant part(s)**	**Condensed tannins (% in dry matter)**		**Free gallic acid (μg GAE/g DW)**		**Gallotannins (μg GAE/g DW)**	
		**Stored**	**Fresh**	**Stored**	**Fresh**	**Stored**	**Fresh**
*Acokanthera oppositifolia*^δ^	Roots	0	0	2.996 ± 2.9963 ns	1.284 ± 1.2841	32.960 ± 0.4281 *	60.355 ± 6.4207
*Artemisia afra*^#^	Aerial parts	0.078 ± 0.0005 ***	0	0	0	76.621 ± 6.4207 ns	97.167 ± 10.7012
*Artemisia afra*^δ^	Aerial parts	0.004 ± 0.0002 *	0	0	0	27.823 ± 11.5573 *	97.167 ± 10.7012
*Buddleja salviifolia*^#^	Leaves	0.011 ± 0.0002 *	0.056 ± 0.0073	29.535 ± 20.1183 ns	0	80.720 ± 17.0557 ns	41.949 ± 1.7122
*Buddleja salviifolia*^#^	Twigs	0.017 ± 0.0047 ns	0.005 ± 0.0050	17.122 ± 3.4244 ns	8.133 ± 5.5646	14.982 ± 8.1329 ns	38.096 ± 17.5500
*Clausena anisata*^#^	Leaves & Twigs	1.394 ± 0.0318 ns	1.329 ± 0.0159	0	0	68.488 ± 5.1366 **	0
*Cussonia spicata*^#^	Leaves	0.012 ± 0.0016 *	0	138.260 ± 41.5208 ns	12.842 ± 12.8415	397.377 ± 55.8931 ns	468.758 ± 81.3346
*Dombeya rotundifolia*^#^	Leaves	1.804 ± 0.0116 **	0.973 ± 0.0529	0	0	41.949 ± 19.6903 ns	0
*Ekebergia capensis*^δ^	Leaves & Twigs	0.654 ± 0.0040 ns	0.523 ± 0.0706	0	0	0 **	19.690 ± 2.5683
*Leonotis intermedia*^δ^	Leaves	0.008 ± 0.0007 **	0	0 ns	3.424 ± 3.4244	17.550 ± 5.5646 ns	11.129 ± 4.2805
*Leonotis leonurus*^δ^	Leaves	0.011 ± 0.0002 ***	0	0 ***	47.085 ± 1.7122	24.827 ± 0.8561 *	5.565 ± 3.8524
*Merwilla plumbea*^δ^	Bulbs	0	0	8.133 ± 8.1329 ns	23.971 ± 7.7049	167.367 ± 13.2695	ND
*Ocotea bullata*^δ^	Bark	1.154 ± 0.0162 **	0.699 ± 0.0354	0	0	68.060 ± 8.9890 *	14.982 ± 7.2768
*Olea europaea*^#^	Leaves	0.010 ± 0.0019 *	0	0	0	127.559 ± 4.2805 ns	121.566 ± 1.7122
*Pittosporum viridiflorum*^#^	Leaves & Twigs	0	0	5.565 ± 5.5646 ns	0	75.337 ± 0.8561 ns	66.776 ± 6.8488
*Plumbago auriculata*^δ^	Leaves	0.003 ± 0.0011 **	0.024 ± 0.0013	3.852 ± 3.8524 ns	0	20.118 ± 5.5646 ns	4.7085 ± 4.7085
*Protorhus longifolia*^δ^	Leaves	0.400 ± 0.0127 ns	0.724 ± 0.0885	2398.787 ± 112.1485 ns	1901.394 ± 137.8318	2726.245 ± 615.9627 ns	4039.926 ± 1368.0443
*Solanum mauritianum*^δ^	Leaves	0.013 ± 0.0013 ns	0.005 ± 0.005	32.103 ± 4.7085 ns	23.971 ± 0.8561	183.047 ± 75.6858	ND
*Spirostachys africana*^#^	Leaves & Twigs	0.348 ± 0.0083 ns	0.365 ± 0.0311	1107.363 ± 228.1501 *	0	2445.016 ± 118.1414 **	16.266 ± 16.2659
*Synadenium cupulare*^#^	Leaves	0.010 ± 0.0002 **	0.004 ± 0.0004	0 ns	8.561 ± 8.5610	20.546 ± 4.2805 ns	54.362 ± 21.8305
*Tetradenia riparia*^δ^	Leaves	0.002 ± 0.0004 ns	0.005 ± 0.0022	0 ***	14.982 ± 0.4280	11.985 ± 1.7122 ns	22.259 ± 13.6976
*Trichilia dregeana*^δ^	Leaves & Twigs	0.198 ± 0.0099 ns	0.138 ± 0.0148	118.998 ± 5.1366 **	0	442.603 ± 65.0634 *	13.270 ± 1.2841
*Ziziphus mucronata*^#^	Leaves	0.008 ± 0.0000 ***	0.046 ± 0.0013	0	0	38.953 ± 12.4134 ns	28.679 ± 1.2842
*Ziziphus mucronata*^δ^	Leaves	0.077 ± 0.0003 **	0.046 ± 0.0013	0	0	47.085 ± 17.1220 ns	28.679 ± 1.2842

ns = not significant; *P* = 0.05 (*); *P* = 0.01 (**); *P* = 0.001 (***); ND = not determined; GAE = Gallic acid equivalents.

^δ^ = Plant material stored for 16 years.

^#^ = Plant material stored for 12 years.

### Antioxidant properties

The effect of long-term storage on the radical scavenging activity of 21 plant materials is presented in Table 3. The lower the EC_50_ value, the higher the antioxidant activity index and the free radical scavenging activity. At 100 μg/ml concentration, the radical scavenging activity of all stored plant materials (with the exception of *Protorhus longifolia*) was either significantly higher or not different when compared to the freshly harvested materials. A comparison based on the EC_50_ values and antioxidant activity indices revealed a significantly higher radical scavenging activity in 58% of the stored plant materials. With the exception of *A. oppositifolia* and *B. salviifolia* (leaves), the radical scavenging activity of the remaining stored plant materials based on their EC_50_ values was not significantly different when compared to the fresh materials. The DPPH radical acts as both the probe and oxidant by accepting electrons from antioxidant compounds in the extract. There is a direct correlation between degree of hydroxylation of the bioactive compounds and DPPH radical scavenging activity [11]. Potent DPPH radical scavenging activities of medicinal plants have also been reported in other studies [11,13,14]. However, the significance of the present study lies in the observed high DPPH radical scavenging activity of aqueous methanol extracts obtained from medicinal plant material after prolonged storage.

**Table 3:** Effect of long-term storage on the free radical scavenging activity of 21 South African medicinal plants

**Plant species**	**Plant part**	**Radical scavenging activity (%) at 100 μg/ml**		**EC**_**50**_**(μg/ml)**		**Antioxidant activity index**	
		**Stored**	**Fresh**	**Stored**	**Fresh**	**Stored**	**Fresh**
*Acokanthera oppositifolia*^δ^	Roots	93.3 ± 0.03 **	92.6 ± 0.10	26.8 ± 2.43 *	18.0 ± 0.34	0.7 ± 0.06 **	1.1 ± 0.02
*Artemisia afra*^#^	Aerial parts	93.8 ± 0.11 *	92.7 ± 0.34	9.3 ± 0.07 ***	12.4 ± 0.15	2.1 ± 0.02 ***	1.6 ± 0.02
*Artemisia afra*^δ^	Aerial parts	94.0 ± 0.07 *	92.7 ± 0.34	6.8 ± 0.50 ***	12.4 ± 0.15	2.9 ± 0.21 **	1.6 ± 0.02
*Buddleja salviifolia*^#^	Leaves	96.2 ± 0.06 ***	93.0 ± 0.40	15.5 ± 0.47 **	10.0 ± 0.61	1.3 ± 0.04 **	2.0 ± 0.12
*Buddleja salviifolia*^#^	Twigs	94.2 ± 0.13 ns	94.3 ± 0.15	17.2 ± 0.32 ns	17.5 ± 0.40	1.1 ± 0.02 ns	1.1 ± 0.03
*Clausena anisata*^#^	Leaves & Twigs	70.8 ± 0.28 ns	72.6 ± 6.21	33.2 ± 3.89 ns	26.8 ± 2.06	0.6 ± 0.07 ns	0.7 ± 0.06
*Cussonia spicata*^#^	Leaves	93.7 ± 0.07 ***	61.6 ± 1.67	14.3 ± 0.22 **	43.6 ± 5.73	1.4 ± 0.02 ***	0.5 ± 0.07
*Dombeya rotundifolia*^#^	Leaves	96.5 ± 0.56 **	93.6 ± 0.27	5.9 ± 0.12 ns	6.1 ± 0.32	3.3 ± 0.07 ns	3.2 ± 0.16
*Ekebergia capensis*^δ^	Leaves & Twigs	94.2 ± 0.42 *	92.8 ± 0.30	4.7 ± 0.37 **	25.5 ± 4.99	4.3 ± 0.32 ***	0.8 ± 0.14
*Leonotis intermedia*^δ^	Leaves	93.3 ± 0.09 *	88.5 ± 1.73	10.6 ± 0.37 ***	51.7 ± 0.32	1.9 ± 0.06 ***	0.4 ± 0.00
*Leonotis leonurus*^δ^	Leaves	93.7 ± 0.18 **	91.6 ± 0.43	16.8 ± 0.06 ***	30.3 ± 0.92	1.2 ± 0.00 ***	0.7 ± 0.02
*Merwilla plumbea*^δ^	Bulbs	8.2 ± 0.61 **	2.6 ± 0.97	ND	ND	ND	ND
*Ocotea bullata*^δ^	Bark	95.0 ± 0.25 **	93.8 ± 0.02	3.2 ± 0.14 **	4.3 ± 0.10	6.3 ± 0.28 **	4.6 ± 0.11
*Olea europaea*^#^	Leaves	94.9 ± 0.20 **	93.2 ± 0.09	14.0 ± 0.48 ***	20.0 ± 0.16	1.4 ± 0.05 ***	1.0 ± 0.01
*Pittosporum viridiflorum*^#^	Leaves & Twigs	93.6 ± 0.10 ns	93.8 ± 0.29	17.9 ± 0.25 ns	17.5 ± 0.27	1.1 ± 0.02 ns	1.1 ± 0.02
*Plumbago auriculata*^δ^	Leaves	50.6 ± 3.97 ns	54.6 ± 1.15	ND	ND	ND	ND
*Protorhus longifolia*^δ^	Leaves	95.8 ± 0.24 **	97.3 ± 0.21	2.2 ± 0.16 ns	2.3 ± 0.14	9.1 ± 0.71 ns	8.5 ± 0.49
*Solanum mauritianum*^δ^	Leaves	34.4 ± 0.73 ***	19.8 ± 1.53	ND	ND	ND	ND
*Spirostachys africana*^#^	Leaves & Twigs	96.6 ± 0.06 ***	91.8 ± 0.34	2.0 ± 0.07 ***	14.4 ± 0.58	10.0 ± 0.35 ***	1.4 ± 0.06
*Synadenium cupulare*^δ^	Leaves	90.9 ± 0.70 ***	46.0 ± 5.30	55.7 ± 0.35	ND	0.4 ± 0.02	ND
*Tetradenia riparia*^δ^	Leaves	68.5 ± 1.39 ***	23.8 ± 2.44	41.0 ± 5.29	ND	0.5 ± 0.06	ND
*Trichilia dregeana*^#^	Leaves & Twigs	95.8 ± 0.46 **	92.3 ± 0.16	5.3 ± 0.02 ***	14.6 ± 0.24	3.7 ± 0.01 ***	1.3 ± 0.02
*Ziziphus mucronata*^#^	Leaves	90.7 ± 0.42 ns	89.0 ± 2.20	29.7 ± 1.02 ns	30.9 ± 1.94	0.7 ± 0.02 ns	0.6 ± 0.04
*Ziziphus mucronata*^δ^	Leaves	91.1 ± 0.18 ns	89.0 ± 2.20	18.1 ± 0.29 **	30.9 ± 1.94	1.1 ± 0.02 ***	0.6 ± 0.04
Ascorbic acid		96.6 ± 0.04		2.1 ± 0.05		9.4 ± 0.23	
Butylated hydroxytoluene		93.2 ± 0.34		3.0 ± 0.04		6.5 ± 0.09	

ns = not significant; *P* = 0.05 (*); *P* = 0.01 (**); *P* = 0.001 (***).

ND = not determined.

^δ^ = Plant material stored for 16 years.

^#^ = Plant material stored for 12 years.

Table 4 presents the effect of long-term storage on the antioxidant activity of medicinal plant materials evaluated based on *β*-carotene bleaching model. The *β*-carotene bleaching assay simulates the oxidation of membrane lipid components and measures antioxidant activity towards linoleic acid [16]. The antioxidant activity of *E. capensis* stored plant material was significantly higher (almost two-fold) compared to the fresh material. On the other hand, the antioxidant activity of *L. intermedia* fresh plant material was significantly higher than that of the stored materials. With the exception of *E. capensis* and *L. intermedia*, there were no significant differences between the antioxidant activities recorded in both the stored and fresh plant materials. The retention of antioxidant activity in stored plant material suggests the stability of bioactive chemicals during prolonged storage. The detected bioactivity in the stored plant material provides interesting prospects in the future development of stable food additive compounds. In previous studies, high antioxidant activity from polar extracts of some plants has been attributed to hydrogen-donating phenolic compounds and flavonoids [2,16]. However, the identification of specific phenolic compounds responsible for the high antioxidant activity of long-term stored plant materials remains a challenge for future research.

**Table 4:** **Effect of long-term storage on antioxidant activity based on*β*-carotene bleaching model and acetylcholinesterase inhibitory properties of 21 South African medicinal plants**

**Plant species**	**Plant part(s)**	**Antioxidant activity (%) at 200 μg/ml**		**AChE inhibition (%) at 1.0 mg/ml**	
		**Stored**	**Fresh**	**Stored**	**Fresh**
*Acokanthera oppositifolia*^δ^	Roots	54.7 ± 3.4 ns	40.0 ± 7.71	81.0 ± 12.11 ns	80.5 ± 1.99
*Artemisia afra*^#^	Aerial parts	45.8 ± 3.34 ns	39.8 ± 4.94	83.2 ± 2.28 ns	89.6 ± 7.44
*Artemisia afra*^δ^	Aerial parts	44.4 ± 7.20 ns	39.8 ± 4.94	89.8 ± 0.57 ns	89.6 ± 7.45
*Buddleja salviifolia*^#^	Leaves	39.1 ± 7.69 ns	58.3 ± 3.04	64.9 ± 11.42 ns	72.5 ± 10.17
*Buddleja salviifolia*^#^	Twigs	58.0 ± 3.92 ns	53.8 ± 8.22	73.0 ± 15.63 ns	63.9 ± 4.05
*Clausena anisata*^#^	Leaves & Twigs	23.6 ± 4.06 ns	49.8 ± 11.19	77.0 ± 6.86 ns	82.2 ± 3.74
*Cussonia spicata*^#^	Leaves	55.7 ± 6.45 ns	41.8 ± 4.70	72.1 ± 12.6 ns	86.5 ± 5.56
*Dombeya rotundifolia*^#^	Leaves	51.8 ± 4.13 ns	58.9 ± 1.40	84.1 ± 5.54 ns	87.6 ± 2.88
*Ekebergia capensis*^δ^	Leaves & Twigs	93.5 ± 7.05 **	52.1 ± 4.97	73.8 ± 7.24 ns	89.7 ± 6.08
*Leonotis intermedia*^δ^	Leaves	32.6 ± 5.34 *	52.9 ± 4.09	68.8 ± 3.12 *	87.8 ± 3.83
*Leonotis leonurus*^δ^	Leaves	40.8 ± 2.32 ns	58.6 ± 7.13	78.1 ± 3.67 ns	73.2 ± 0.43
*Merwilla plumbea*^δ^	Bulbs	57.0 ± 6.42 ns	45.1 ± 4.06	58.7 ± 6.52 ns	81.5 ± 2.11
*Ocotea bullata*^δ^	Bark	57.8 ± 7.33 ns	62.3 ± 8.83	84.8 ± 3.98 ns	87.1 ± 2.63
*Olea europaea*^#^	Leaves	48.8 ± 2.84 ns	48.2 ± 0.59	69.2 ± 5.99 ns	85.4 ± 3.39
*Pittosporum viridiflorum*^#^	Leaves & Twigs	62.9 ± 6.65 ns	39.1 ± 6.80	96.2 ± 0.71 ns	70.5 ± 8.36
*Plumbago auriculata*^δ^	Leaves	62.2 ± 10.87 ns	52.8 ± 1.99	82.3 ± 5.54 ns	87.3 ± 2.20
*Protorhus longifolia*^δ^	Leaves	90.9 ± 8.88 ns	72.9 ± 2.62	51.8 ± 9.07 ns	40.07 ± 2.60
*Solanum mauritianum*^δ^	Leaves	38.9 ± 10.07 ns	49.4 ± 4.92	78.5 ± 5.84 ns	85.9 ± 3.94
*Spirostachys africana*^#^	Leaves & Twigs	62.1 ± 4.40 ns	58.3 ± 3.24	90.4 ± 5.57 ns	82.4 ± 3.51
*Synadenium cupulare*^δ^	Leaves	54.5 ± 5.06 ns	45.3 ± 2.04	75.3 ± 4.07 ns	81.1 ± 2.77
*Tetradenia riparia*^δ^	Leaves	67.2 ± 4.89 ns	64.5 ± 8.38	80.8 ± 1.73 *	65.4 ± 4.85
*Trichilia dregeana*^#^	Leaves & Twigs	65.2 ± 7.46 ns	50.6 ± 8.81	94.8 ± 2.82 *	81.1 ± 3.99
*Ziziphus mucronata*^#^	Leaves	54.5 ± 3.65 ns	42.6 ± 6.62	84.8 ± 6.78 ns	87.2 ± 10.04
*Ziziphus mucronata*^δ^	Leaves	24.1 ± 11.13 ns	42.6 ± 6.62	90.4 ± 4.09 ns	87.2 ± 10.04
Galanthamine				84.1 ± 1.45	
Butylated hydroxytoluene		94.5 ± 1.71			

ns = not significant; *P* = 0.05 (*); *P* = 0.01 (**); *P* = 0.001 (***).

^δ^ = Plant material stored for 16 years.

^#^ = Plant material stored for 12 years.

Galanthamine (20 μM) was used as a positive control in acetylcholinesterase assay.

### Acetylcholinesterase inhibition activity

Table 4 presents the effect of long-term storage on AChE inhibitory properties of the evaluated plant materials. Stored plant materials of *T. riparia* and *T. dregeana* showed a significantly higher AChE inhibition than the fresh ones. There was no significant difference between the percentage AChE inhibition by the stored and fresh materials of the remaining plant species. In general, the evaluated plant species exhibited high AChE inhibitory activity. Interestingly, medicinal plant materials retained AChE inhibitory activity even after prolonged storage (12 or 16 years). The results of the present study confirm the therapeutic value of stored medicinal plants in the pharmacotherapy of AD disease. The AChE inhibitory properties of plant-derived extracts obtained from freshly harvested material have been previously reported [16,32]. Recent studies have demonstrated a direct association between AD and antioxidant activity [16]. However, this is the first report on the antioxidant and AChE inhibitory properties of long-term stored medicinal plants. The present findings are important for traditional systems which are characterised by an holistic approach to health provision, based on the prophylactic properties of medicinal plants [6].

## Conclusions

The current study presents evidence that dried medicinal plants stored under dark conditions at room temperature remain biologically active after long-term storage. Extracts of the stored plant material still exhibited potent antioxidant and AChE-inhibitory properties. These findings are significant as some medicinal plants may be utilised long after their time of harvesting. In addition, the prevention strategies practised in the Ayurvedic, Chinese and African medicinal systems often involve regular intake of medicinal plant extracts and/or herbal preparations, which are responsible for counteracting the oxidative stress effects caused by ROS. The high antioxidant activity and stability of the bioactive compounds in these medicinal plants offer interesting prospects for the identification of novel principles for application in food and pharmaceutical formulations. However, *in vitro* and *in vivo* safety evaluation of the stored medicinal plants is required.

## Abbreviations

ACh, Acetylcholine; AChE, Acetylcholinesterase; AD, Alzheimer’s disease; AAI, Antioxidant activity index; BHT, Butylated hydroxytoluene; CE, Catechin equivalents; DPPH, 2,2-diphenyl-1-picrylhydrazyl; DTNB, 5,5-dithiobis-2-nitrobenzoic acid; DW, Dry weight; GAE, Gallic acid equivalents; HE, Harpagoside equivalents; RSA, Radical scavenging activity; ROS, Reactive oxygen species.

## Competing interests

The authors declare that they have no competing interests.

## Authors’ contributions

SOA participated in the collection of plant materials, study design, extraction, conducting the assays, statistical analysis and drafting the manuscript. AOA was involved in the collection of plant materials, study design, extraction, carrying out the assays and editing the manuscript. MM participated in the collection of plant materials, study design, extraction, conducting the assays and drafting the manuscript. JVS coordinated the storage of plant materials and revised the manuscript. All authors read and approved the final manuscript.

## Pre-publication history

The pre-publication history for this paper can be accessed here:

http://www.biomedcentral.com/1472-6882/12/87/prepub
